# Long-Term Effects of Residual Chlorine on *Pseudomonas aeruginosa* in Simulated Drinking Water Fed With Low AOC Medium

**DOI:** 10.3389/fmicb.2018.00879

**Published:** 2018-05-03

**Authors:** Guannan Mao, Yuhao Song, Mark Bartlam, Yingying Wang

**Affiliations:** ^1^Key Laboratory of Pollution Processes and Environmental Criteria (Ministry of Education), Tianjin Key Laboratory of Environmental Remediation and Pollution Control, College of Environmental Science and Engineering, Nankai University, Tianjin, China; ^2^State Key Laboratory of Medicinal Chemical Biology, Nankai University, Tianjin, China; ^3^College of Life Sciences, Nankai University, Tianjin, China

**Keywords:** chlorination, regrowth, antibiotic-resistant, *Pseudomonas aeruginosa*, low AOC medium

## Abstract

Residual chlorine is often required to remain present in public drinking water supplies during distribution to ensure water quality. It is essential to understand how bacteria respond to long-term chlorine exposure, especially with the presence of assimilable organic carbon (AOC). This study aimed to investigate the effects of chlorination on *Pseudomonas aeruginosa* in low AOC medium by both conventional plating and culture-independent methods including flow cytometry (FCM) and quantitative PCR (qPCR). In a simulated chlorinated system using a bioreactor, membrane damage and DNA damage were measured by FCM fluorescence fingerprint. The results indicated membrane permeability occurred prior to DNA damage in response to chlorination. A regrowth of *P. aeruginosa* was observed when the free chlorine concentration was below 0.3 mg/L. The bacterial response to long-term exposure to a constant low level of free chlorine (0.3 mg/L) was subsequently studied in detail. Both FCM and qPCR data showed a substantial reduction during initial exposure (0–16 h), followed by a plateau where the cell concentration remained stable (16–76 h), until finally all bacteria were inactivated with subsequent continuous chlorine exposure (76–124 h). The results showed three-stage inactivation kinetics for *P. aeruginosa* at a low chlorine level with extended exposure time: an initial fast inactivation stage, a relatively stable middle stage, and a final stage with a slower rate than the initial stage. A series of antibiotic resistance tests suggested long-term exposure to low chlorine level led to the selection of antibiotic-resistant *P. aeruginosa*. The combined results suggest that depletion of residual chlorine in low AOC medium systems could reactivate *P. aeruginosa*, leading to a possible threat to drinking water safety.

## Introduction

Safe and clean water is a basic necessity that is essential for all aspects of everyday life. Nowadays, residual disinfectants are crucial to inhibit microbial growth and maintain water quality throughout distribution until the point-of-use in many countries (Proctor and Hammes, 2015; Prest et al., 2016). Among the many various disinfection methods, chlorination is widely used in public drinking water supply systems due to its low cost and simple use (Levy et al., 2014). The residual chlorine levels may be depleted over long distances or within complicated drinking water distribution systems and, as a consequence, excessive bacterial growth may lead to deterioration of water quality (Nescerecka et al., 2014). It has been reported that insufficient chlorine levels could result in a higher proportion of intact cells in chlorinated systems (Niquette et al., 2001; Gillespie et al., 2014). Meanwhile, previous studies have also found that biological stability is associated with nutrient limitation in drinking water distribution networks without any residual disinfectants (Vital et al., 2012; Lautenschlager et al., 2013). However, it is difficult to maintain low levels of nutrients during the distribution of drinking water. For instance, organic compounds from pipes have been shown to migrate into the water phase, which may not only support microbial growth (Wen et al., 2015) but also react with chlorine. Hence, the presence of nutrients and an inadequate chlorine concentration in drinking water would stimulate bacterial proliferation. Therefore, it is essential to investigate how microorganisms respond to real chlorinated drinking water systems with the presence of dissolved organic matter.

Previous studies on the effects of disinfectant on pure cultures have relied on conventional plating methods to quantify the extent of inactivation (Delaedt et al., 2008; Francisque et al., 2009; Huang et al., 2011). However, the culture-dependent method heterotrophic plate counts (HPC) has clear limitations, with only a minority of viable cells able to be cultivated in drinking water (Hammes et al., 2008). Culture-independent methods, such as flow cytometry (FCM) and quantitative PCR (qPCR), are therefore used for assessment and monitoring of indigenous bacteria in drinking water (McDaniels et al., 2005; Hammes et al., 2008; Wang et al., 2010b; Douterelo et al., 2014; Besmer et al., 2017). The combination of FCM and fluorescent stains has been successfully applied to the analysis of non-chlorinated drinking water (Besmer et al., 2014) and drinking water with residual chlorine (Gillespie et al., 2014). Bacteria in water samples can be enumerated by qPCR based on the quantification of target gene copies (Douterelo et al., 2014). The culture-independent methods are therefore conducive to providing more realistic information than conventional culture-dependent methods.

*Pseudomonas aeruginosa* is a rod-shaped Gram-negative opportunistic pathogen that may cause human infections, and *P. aeruginosa* is also considered to be the most important pseudomonad in drinking water (Mena and Gerba, 2009). Previous studies have reported that *P. aeruginosa* could develop resistance to low levels of chlorine used in water treatment (Ridgway and Olson, 1982; Shrivastava et al., 2004). Furthermore, *P. aeruginosa* is able to grow under a wide variety of environmental conditions, especially in low-nutrient water (Silva et al., 2008). Mendis et al. (2014) found *P. aeruginosa* growing in nutrient-poor water exhibited considerably diverse properties compared to bacteria grown in rich media under laboratory conditions. Additionally, several opportunistic pathogens have been shown to reactivate during long-term storage of added chlorine (Jjemba et al., 2010). Therefore, it is essential to investigate the dynamics of *P. aeruginosa* subjected to long-term chlorine disinfection in drinking water distribution systems.

In the present study, we used culture-independent methods (i.e., FCM and qPCR) as well as culture-dependent methods (i.e., HPC) to investigate the effects of long-term low levels of chlorine on *P. aeruginosa* growth in bioreactor fed with low assimilable organic carbon (AOC) medium. The aims of this study were to: (i) determine the threshold chlorine concentration in drinking water in the presence of nutrients; (ii) investigate whether long-term exposure of chlorine leads to selection of chlorine-resistant *P. aeruginosa*; and (iii) elucidate the inactivation kinetics for long-term chlorination.

## Materials and Methods

### Microorganism and Pre-cultivation

*Pseudomonas aeruginosa* PAO1 was incubated in sterile Luria-Bertani (LB) broth overnight at 37°C. *P. aeruginosa* was streaked onto the LB agar plate and grown for 24 h at 37°C. A single colony was transferred with a loop into the sterile 100-times diluted LB broth and incubated for 24 h at 37°C on a shaking incubator to be used as inoculum.

### Low AOC Medium Systems

A bioreactor (BioFlo CelliGen 115, New Brunswick, Eppendorf, United States) was used to simulate low AOC medium systems. The 10000-times diluted LB medium was used to simulate the low AOC medium system. The method of synthetic substrates addition has been successfully applied to analyze the effects of nutrients on the regrowth bacteria in drinking water distribution system (Van der Kooij et al., 1982; Miettinen et al., 1999; Ellis et al., 2000; Jegatheesan et al., 2004; Tsai, 2005; Oh et al., 2009). Sterile 10000-times diluted LB medium (simulated drinking water) was continuously pumped into the bioreactor (flow rate 2 ± 0.07 mL/h) and the mixed water was pumped out of the bioreactor with two peristaltic pumps (maintaining a constant water level in the bioreactor). The bioreactor was cleaned with soap and rinsed with deionized water twice and then air-dried. The bioreactor was sterilized by autoclaving. The bioreactor was supplied with sterile 1 L 10000-times diluted LB medium and sealed by headplate to avoid bacterial contamination. The main parameters of the medium (i.e., temperature, 23.1 ± 0.35°C and pH, 7.46 ± 0.07) were controlled and monitored by bioreactor. The chlorine solution was obtained by adding a sodium hypochlorite (NaOCl) solution with a concentration of about 14.5% active chlorine (Energy Chemical, China) to the carbon-free deionized water to the final concentration of 20 mg/L. Chlorine solution was pumped into the bioreactor by a peristaltic pump (the flow rate see below).

### Chlorination Experiments With *P. aeruginosa*

For *P. aeruginosa* disinfection experiments, *P. aeruginosa* was added to the bioreactor with a sterile syringe. When the final cell concentration reached 10^7^ cells/mL, chlorine solution was pumped into the bioreactor. The concentration of chlorine was increased from 0 mg/L to 0.47 mg/L (flow rate 2 ± 0.07 mL/h) in the first 10 h. When chlorine peaked at 0.47 mg/L, the addition of chlorine was stopped. The experiment was stopped when the concentration of chlorine was under 0.02 mg/L. Samples were taken from system at regular time interval. Free chlorine concentration was measured immediately after sampling. Cultivable cells were measured with HPC (as described below). FCM was applied in total cell and intact cell number analysis. Quenching step was not included in this study since it had no significant effect on cell counting (**Supplementary Figure S1**) with the chlorine concentration applied in the current study. All measurements were performed in triplicate.

### Long-Term Chlorination Experiments on Constant Concentration

The sterilized, 10,000-times diluted LB medium was spiked with *P. aeruginosa* (final concentration of 10^7^ cells/mL) and chlorine. The concentration of chlorine was maintained constant at 0.3 mg/L by an uninterrupted supply of fresh chlorine (flow rate 0.9 ± 0.06 mL/h). 124 h of chlorination was performed. Samples were taken at regular time intervals and collected in 40 mL sterile vials. Antibiotic resistance, FCM and qPCR analysis were carried out after sampling (as described below). All measurements were performed in triplicate.

The chlorination inactivation kinetics were analyzed based on plotting log(*N*/*N*_0_) versus the chlorination exposure according to the equation log(*N*/*N*_0_) = –k[*Ct*]. [*N*] is the intact cell number, and [*N*_0_] is the initial cell concentration. [*Ct*]: Chlorine concentration [*C*] multiplied by the exposure time [*t*] for each time point. The rate constant [*k*] is determined by fitting log(*N*/*N*_0_) against [*Ct*].

### Chlorine Determination

The chlorine concentrations were determined by the *N,N*-diethyl-*p*-phenylenediamine (DPD) colorimetric method using a pocket colorimeter (Hach-Lange, Salford, United Kingdom). In this method, the DPD powder is added to the sample and oxidized by chlorine. The solution becomes red in color and the color intensity is proportional to the chlorine concentration. The range of the colorimeter is 0.02 to 8 mg/L. The colorimeter has an accuracy of ±3%.

### FCM Measurements

Flow cytometry was performed on a Partec CyFlow Space flow cytometer (Partec GmbH, Münster, Germany) with a 50 mW blue solid-state laser, emitting at a fixed wavelength of 488 nm. Detection was triggered on green fluorescence and collected as logarithmic signals. Green fluorescence was collected at FL1 = 520 ± 20 nm, and red fluorescence was collected at FL3 = 630 nm, while forward and sideward scattered light intensities were collected as well. The instrument is equipped with volumetric counting hardware. In short, the number of particles in 200 μL of each sample were automatically enumerated (Berney et al., 2008). Counts quantification limited of the instrument were 1000 cells/mL and the instrument have an instrumental error below 5% (Hammes et al., 2008; Lee et al., 2016). The FCM regularly maintains and calibrate with calibration beads. The corresponding light signals are then converted to electronic signals and data were analyzed with the Flomax software.

For measurements of total cell count (TCC), 10 μL SYBR Green I (SGI) (1:100 dilution in dimethyl sulfoxide (DMSO), Invitrogen) was added to 1 mL sample (dilutions of the sample were performed when appropriate). Samples were briefly vortexed and incubated at 37°C for 15 min in the dark before measurement. Strictly controlling staining time avoid interference of DMSO solvent on viability of cells (Berney et al., 2008; Nescerecka et al., 2016a). For measurements of intact cell count (ICC), Propidium Iodide (PI) (30 mW, Invitrogen) was combined with SYBR Green I stock at a ratio of 1:50. The same protocol as described above was used for the staining.

### Plate Counts and Antibiotic Resistance Testing

To obtain HPC, samples were diluted 10-times with sterile physiological solution (0.85% NaCl) and 100 μL of the appropriate dilutions were spread onto the LB agar plates. Colony-Forming Units (CFUs) were counted after 24 h incubation at 37°C.

Samples were tested for antibiotic resistance using the National Food and Drug Administration disk diffusion method. The following antibiotic disks were tested: ciprofloxacin (5 μg/disk), gentamicin (10 μg/disk), erythromycin (15 μg/disk), norfloxacin (10 μg/disk), and tetracycline (30 μg/disk). Samples were streaked on the MH agar plate containing antibiotic disks, and the diameter (*D*) of the inhibition zone was measured after 18 h incubation at 37°C.

### DNA Extraction and Quantitative PCR

Total DNA was extracted from each sample isolate by DNA extraction kit (Qiagen, Germany) according to the manufacturer’s instructions. For amplification of the highly conserved region of *gyrA* gene of *P. aeruginosa*, the forward primer (5′-TTATGCCATGAGCGAGCTGGGCAACGACT-3′) and reverse primer (5′-AACCGTTGACCAGCAGGTTGGGAATCTT-3′) (Kureishi et al., 1994) were used. Each 20 μL reaction mixture contained 10 μL of 2 × GoTaq^®^ qPCR Master Mix (Promega, United States), 0.05 μL of each primer, 7.2 μL nuclease-free water, and 2 μL DNA templates. The cycling programs were optimized at 95°C for 420 s for preincubation, followed by 3-step amplification with 50 cycles of 95°C for 15 s, 55°C for 30 s and 72°C for 30 s with fluorescence acquisition. The melting curve was generated at the end. In every run, standard curves were plotted using a 10-fold serial dilution of purified plasmid DNA containing the target insert. Negative controls contained nuclease-free water instead of the DNA template. All samples were run in triplicate.

## Results

### Change of *P. aeruginosa* Cells During the Chlorination Process

**FIGURE 1:**
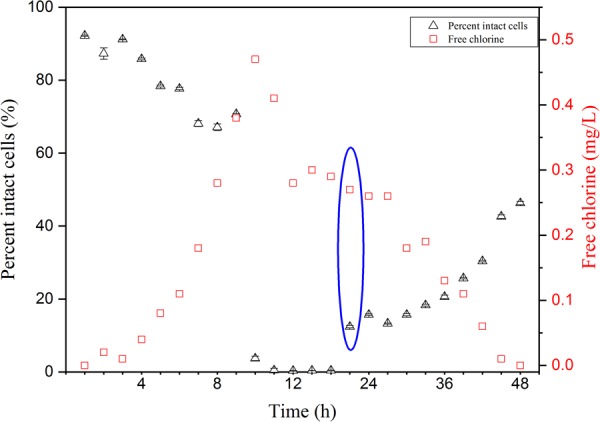
Free chlorine concentration changes and proportions of intact cells of *Pseudomonas aeruginosa* over 48 h of chlorination in the simulated low assimilable organic carbon (AOC) medium systems. SGI/PI stained cells were enumerated by FCM for intact cell number. Bacteria reactivation was observed from blue cycle data.

**FIGURE 2:**
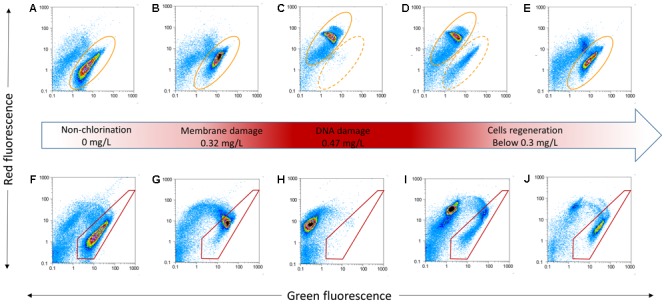
Examples of flow cytometric dot plots of *P. aeruginosa* for TCC stained with SGI **(A–E)** and ICC stained with SGI/PI **(F–J)** and various status (middle red arrow). Yellow ellipses (dashed and solid) tracked SGI fingerprint shift due to different chlorination. Red polygons illustrated membrane damage with change of chlorine.

Cell damage in chlorinated water with AOC concentration was monitored over 48 h. Free chlorine concentrations increased from 0 to 0.47 mg/L in the first 10 h, then decreased to below 0.02 mg/L in this study (**Figure 1**). Four distinct phases (i.e., non-chlorinated; membrane damage; DNA damage and cell regeneration) were revealed during the chlorination process (**Figure 2**). **Figures 2A–E** shows the result of samples stained with SGI. The TCC of 1.3 × 10^7^ cells/mL was observed before chlorination, as shown in the yellow ellipse gate (**Figure 2A**). In general, the TCC gate (or so-called fluorescence fingerprint) is fixed when SGI stains all cells indiscriminate of viability (Berney et al., 2008; Prest et al., 2013). However, Lee et al. (2016) found the fixed-gate strategy was incorrect for measurement of TCC when DNA underwent considerable damage. In this study, the shift of the gate [yellow dashed ellipse shifted to yellow solid ellipse (**Figure 2C**)] was also observed at high chlorine concentration (0.47 mg/L) after 1 h. If the fixed-gate strategy is used for quantification, the TCC decreased dramatically. This is important as it indicated bacteria experienced considerable DNA damage (e.g., DNA unwinding) at the specific chlorine dose of 0.47 mg/L. New dots within the yellow dashed ellipse appeared when chlorine was gradually depleted (below 0.3 mg/L) (**Figure 2D**). It can be concluded from these results that the cells begin to recover (regrow) when the free chlorine residual level dropped below 0.3 mg/L. After 12 h (hours 36–48) of suboptimal chlorination levels, the TCC fingerprint (yellow solid ellipse) was restored to the same position as the original (**Figures 2E** vs. **2A**).

During the period of increasing chlorine, the percentage of intact cells decreased slightly in the first 9 h (**Figure 1**). When the free residual chlorine level approached 0.47 mg/L, the proportion of intact cells dropped dramatically to less than 1%. From the FCM plots, various cellular changes can be observed at different concentrations of chlorine. **Figures 2F–J** shows cells that are stained with a combination of SGI and PI. The cells take up red fluorescent PI when their membrane integrity is lost (Berney et al., 2007). The ICC data showed cell damage (**Figures 2F–H**) due to increasing chlorine concentration and cell regeneration (**Figures 2I,J**) as a result of insufficient chlorination. It is evident that membrane damage (**Figure 2G**) could be detected at 0.32 mg/L-Cl. After a higher exposure level (0.47 mg/L; 1 h, **Figure 2H**), most of the *P. aeruginosa* cells had lost their integrity, while at the same time DNA damage was also observed via the changing gate of TCC. The results demonstrated that chlorine first damaged the cell membranes and then attacked intracellular components such as the chromosome. It is interesting to note that at a disinfection residual below 0.3 mg/L free chlorine, bacterial regrowth was detected in this system (**Figure 1**, blue circle). The intact region became dominant once again on the FCM plots (**Figures 2I,J**). This indicates that part of the population had the ability to regenerate in the presence of residual chlorine.

**FIGURE 3:**
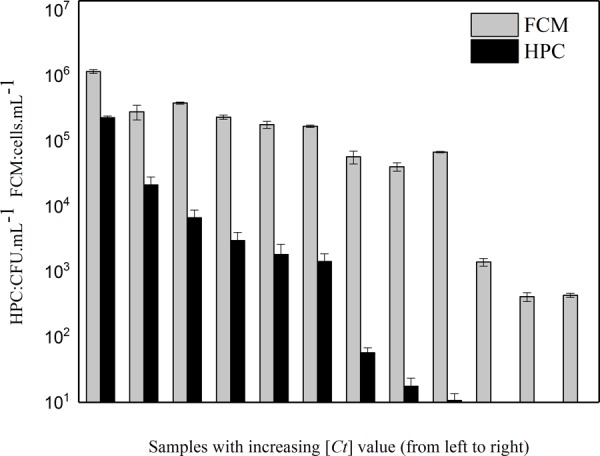
Relative decrease of intact cell number determined by two different methods (i.e., culture-dependent method HPC and culture-independent method FCM) with increasing [*Ct*] value in the simulated low AOC medium systems.

Intact cells in *P. aeruginosa* was also enumerated by HPC. Both microbiological parameters (FCM and HPC) showed a decrease with increasing [*Ct*] value (**Figure 3**). However, the conventional HPC results were lower than those measured by FCM, indicating some *P. aeruginosa* entered a viable-but-not-cultivable (VBNC) bacterial state after disinfection treatment. The percentage of cells in the VBNC state increased along with increasing chlorine concentration (**Figure 3**).

### Effects of Long-Term Exposure to Constant Chlorine on *P. aeruginosa*

#### Temporal Change of Cell Concentrations Analyzed by FCM and qPCR

**FIGURE 4:**
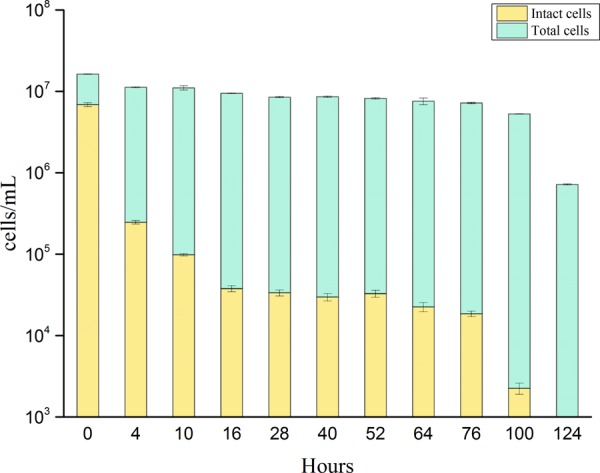
Changes of total cell concentration (stained by SGI) and intact cell concentration (stained by SGI/PI) of *P. aeruginosa* during long-term (124 h) exposure to chlorine at a constant chlorine concentration of 0.3 ± 0.05 mg/L. Error bars indicate standard deviation of triplicate measurements.

A regrowth threshold (0.3 mg/L-Cl) was observed during the chlorination process. Long-term (i.e., 124 h) exposure to this constant chlorine concentration (0.3 ± 0.05 mg/L) revealed temporal changes in TCC and ICC (**Figure 4**). During the initial 10 h of exposure, the ICC decreased substantially from 6.8 × 10^6^ cells/mL to 9.8 × 10^4^ cells/mL, indicating that most bacterial membranes were damaged during initial exposure to chlorine. The number of intact cells remained stable between 16 and 76 h after exposure, showing that some of the population gained resistance to chlorine exposure. Finally, all of the *P. aeruginosa* cells were inactivated after prolonged exposure to chlorine. The TCC remained constant over 100 h. Due to oxidative damage of DNA during long-term exposure, the fluorescence intensity weakened slightly (Phe et al., 2004). Lastly the final concentration of total bacteria decreased to 7.1 × 10^5^ cells/mL. At a concentration of 0.3 mg/L, there was no apparent resuscitation over 124 h with long-term chlorine exposure.

**FIGURE 5:**
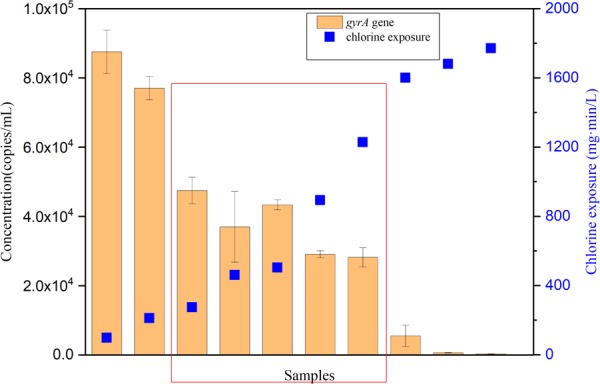
The variation tendency of *gyrA* gene with increasing chlorine exposure in the simulated low AOC medium systems. Red region indicates a relative stable period of *gyrA* gene during chlorine disinfection. Error bars indicate standard deviation of triplicate measurements.

Temporal changes of *P. aeruginosa* during long-term exposure to chlorine was also monitored with qPCR. *gyrA* encodes for the subunit A of DNA gyrase in *P. aeruginosa* (Schwartz et al., 2006). Serial dilutions of the plasmid containing *gyrA* gene were used to establish the standard curve (**Supplementary Figure S2**), ranging from 1 × 10^3^ – 1 × 10^9^ copies per reaction. The standard curve slope was –3.39 with a correlation coefficient greater than 0.99. The qPCR efficiency was 97%. The copies of the *gyrA* gene of *P. aeruginosa* during chlorine disinfection were monitored by a culture-independent method (qPCR) (**Figure 5**). At an initial chlorine exposure of 98 mg⋅min/L the *gyrA* copies were 8.8 × 10^4^ copies/mL. Afterwards, the number of *gyrA* copies decreased to 3.7 × 10^4^ copies/mL at a chlorine exposure of 460 mg⋅min/L. With increasing exposure to chlorine, it can be observed that in the red region, the concentration of *gyrA* remained stable or even increased rather than continuously decreased, which is consistent with the FCM results. The concentration of *gyrA* was negligible at higher exposures (1700 mg⋅min/L), presumably due to further oxidative damage of the target gene. The *gyrA* gene abundance showed a similar trend to the FCM method: (i) a strong reduction of concentration during the initial exposure stage, and (ii) a relatively stable period from 16 to 76 h of exposure.

#### Kinetics of Chlorine Disinfection

**FIGURE 6:**
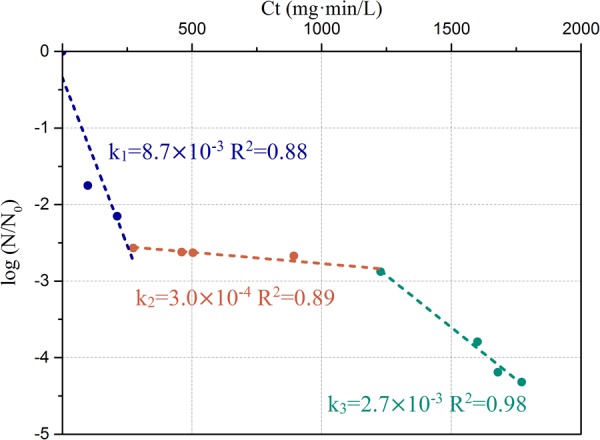
Chlorine inactivation kinetics of *P. aeruginosa* in the simulated low AOC medium systems. The slopes and corresponding *R*^2^-value are labeled on the figure.

In the simulated drinking water system with a constant concentration of 0.3 ± 0.05 mg/L-Cl, the kinetics of *P. aeruginosa* inactivation can be characterized by three stages (**Figure 6**). During the initial chlorine exposure, between 0 and 270 mg⋅min/L of [*Ct*], the first stage led to 2.5-log removal of live bacteria. The ratio of inactivation kinetics decreased strongly with a slope of 8.7 × 10^-3^ L/(mg⋅min). This indicated that most of the cells were heavily damaged or lysed. For 270 < [*Ct*] < 1200 mg⋅min/L, a plateau was reached. The slope of the second stage [3.0 × 10^-4^ L/(mg⋅min)] was much lower than that of the first stage. The kinetic change indicated development of chlorine resistance in bacterial community. For the higher [*Ct*] values, the third stage [2.7 × 10^-3^ L/(mg⋅min)] showed a slight decay again with further deterioration of the environment. However, the slope [*k*] of the third stage is lower than that of the first stage.

#### Antibiotic Resistance

The sensitivity of *P. aeruginosa* to different antibiotics was estimated during the long-term chlorine exposure. The antibiotic resistance of *P. aeruginosa* changed with increasing disinfectant exposure time. **Table 1** lists the diameter of the inhibition zone and antibiotic sensitivity profiles of *P. aeruginosa* at the different exposure times to 0.3 mg/L chlorine. The experimental results showed the selection of quinolone-resistant *P. aeruginosa* in the system treated with chlorine. Specifically, *P. aeruginosa* cells developed resistance to ciprofloxacin and norfloxacin after 52 h of exposure. However, after 64 h, all of the cells had already lost their cultivability.

**Table 1:** Diameter of inhibition zone and sensitivity of *P. aeruginosa* to different antibiotics at different exposure time during long-term exposure to 0.3 mg/L chlorine.

Time (H)	Diameter of inhibition zone (mm)				
	Ciprofloxacin	Gentamicin	Erythromycin	Norfloxacin	Tetracycline
0	18(S)	18(S)	2(R)	16(S)	16(S)
4	22(S)	20(S)	0(R)	16(S)	16(S)
10	22(S)	20(S)	2(R)	18(S)	18(S)
16	20(S)	16(S)	0(R)	16(S)	18(S)
28	22(S)	18(S)	2(R)	18(S)	20(S)
40	22(S)	18(S)	2(R)	20(S)	18(S)
52	8(R)	16(S)	0(R)	6(R)	16(S)
64	14(R)	18(S)	0(R)	12(R)	20(S)
76	N	N	N	N	N
100	N	N	N	N	N
124	N	N	N	N	N

*S, sensitive; R, resistive; N, not detected.*

## Discussion

Chlorination is the most common used disinfection for the bacteriological quality control of drinking water distribution systems. To ensure the water quality, residual disinfections were required to remain during distribution in some countries. Previous studies have already confirmed insufficient chlorine levels could result in regrowth bacteria in chlorinated systems (Niquette et al., 2001; Gillespie et al., 2014). However, the microorganisms respond to inadequate chlorinated water in low AOC medium for long-term exposure, which was not previously possible. In the present study, application of FCM combined with different fluorescent stains (SGI and SGI/PI) was used to rapidly assess not only the effects of disinfection, but also changes in the bacteria due to suboptimal chlorine levels in low AOC medium (less than 0.50 mg/L). FCM in combination with SGI and PI is a highly conservative method that distinguishes between viable and non-viable cells based on membrane integrity (Ramseier et al., 2011). For instance, Berney et al. (2008) showed the last viability indicator was the membrane integrity when *Escherichia coli* was exposed to UV-A disinfection. Our results showed that substantial inactivation of bacteria was found when free chlorine levels reached 0.47 mg/L, which is consistent with an earlier report (Gillespie et al., 2014).

In the current study, changes in cell membrane permeability and DNA damage during chlorine exposure were reflected by FCM analysis. Furthermore, flow cytometric TCC fingerprints demonstrated considerable damage to DNA at a specific chlorine dose. Similar TCC and ICC fingerprint results were observed during ozonation of *E. coli* (Lee et al., 2016). Comparison between TCC and ICC fingerprints provided evidence that membrane permeability occurred prior to DNA damage. Such membrane damage would lead to considerable release of ATP from bacteria (Nescerecka et al., 2016b). Francisque et al. (2009) also found that a residual chlorine level of less than 0.3 mg/L was insufficient to inhibit bacterial growth. This is most likely due to the cells being partially damaged and/or developing resistance to chlorine. In addition, the presence of an appropriate amount of nutrients available in this system provides important support for cell regrowth. Meier and Bendinger (2016) found an elevated nutrient condition in water could increase the regrowth of *P. aeruginosa* after chlorination. A free residual chlorine level ≥ 0.3 mg/L should be maintained at the exit of the water treatment plant (USEPA, 1989; WHO, 1997). Based on the results of cellular changes of *P. aeruginosa* during the chlorination process, a free chlorine concentration of 0.3 mg/L was chosen for the following long-term exposure experiments. It is important to note that water quality parameters including pH and temperature (constant in this study) could influence disinfection efficacy (Meier and Bendinger, 2016). Biofilms occur usually in surfaces which are in contact with water even disinfectant residual water. The formation of biofilm will protect the bacteria from chlorine disinfection (Pintar and Slawson, 2003).

Combination FCM and qPCR methods to reveal temporal changes of bacterial cells during long-term exposure to constant inadequate chlorine concentration in low AOC medium. Overall, the chlorination of *P. aeruginosa* in low AOC medium could be characterized by three stages (**Figure 6**). The rate of inactivation of the initial stage was relatively fast, followed by a stable plateau stage and then finally damaged as indicated by a lower slope. The first two stages have been reported in previous studies during disinfection of *Mycobacterium avium* (Luh and Marinas, 2007) and chlorination of drinking water (Ramseier et al., 2011). Compared to the two stages of disinfection in previous studies, our results showed a more complex inactivation mechanism. This may be attributed to the longer chlorine exposure time than in previous reports. Meanwhile, the dose of chlorination applied here is relatively low compared to previous studies. Such long-term exposure to low residual chlorine revealed subtle changes at the cellular level. Furthermore, free chlorine and AOC are the two main factors regulating the bacterial regrowth in drinking water distribution systems where chlorine is applied as a disinfectant (Liu et al., 2015). Here we argue that a more detailed inactivation mechanism was revealed partly due to the fact free chlorine was the only influencing factor for the inactivation kinetics since a constant level of AOC was applied in the present system. To the best of our knowledge, this is the first demonstration of three stages of inactivation kinetics for *P. aeruginosa* with chlorine disinfection. The results demonstrated a selection of chlorine resistant cells during long-term exposure. Similar findings were reported in which chlorine-tolerant microbes were selected from chlorinated water (Ridgway and Olson, 1982; Shrivastava et al., 2004; Chiao et al., 2014). Compared to previous studies, a relatively low rate constant of 99% inactivation was observed in this study (**Table 2**). Culture-dependent methods applied in previous studies may lead to significant underestimation of bacterial counts when bacteria enter the “VBNC” state, resulting in a higher inactivation rate constant. Various combinations of treatments (exposure time and concentration of chlorine) may result in different inactivation kinetics even at the same [*Ct*] value. It has previously been demonstrated that a higher concentration of chlorine with a shorter exposure time is more beneficial for inactivation (Huang et al., 2011). In our low AOC medium system, *P. aeruginosa* may grow attached to surface and formed the biofilms. The biofilms with the extracellular polymeric substance (EPS) could reduce efficiency of chlorine disinfection and also lead to small inactivation rate constants (Liu et al., 2016; Shen et al., 2016). Overall, the results showed that *P. aeruginosa* could resist a longer exposure time with a lower dose of chlorine. It is thus inferred that long-term storage of water with residual chlorine may be advantageous for the prevalence of chlorine-resistant bacteria. To avoid this problem, such as decreasing storage times or flush 30 s tap water before first use long-term storage of water are required.

**Table 2:** The rate constants k to achieve 99% inactivation of different microorganisms with chlorine disinfection.

Microorganism	*k*_99%_ (L/mg⋅min)	Reference
*P. aeruginosa* (23°C; pH 7.5)	8.7 × 10^-3^	This study
*P. aeruginosa* (22°C)	50.22 (max inactivation rate)	Xue et al., 2013
*E. coli* (25°C; pH 7.5)	4.35	Helbling and VanBriesen, 2008
HNA (22°C; pH 8)	1.1	Ramseier et al., 2011
LNA (22°C; pH 8)	1.0 × 10^-1^	Ramseier et al., 2011

Previous studies have revealed the selection pressure of chlorination for antibiotic-resistance bacteria (Shrivastava et al., 2004; Huang et al., 2011), and our results further indicated that long-term exposure with low chlorine concentration (simulated stagnation) could elevate resistance of *P. aeruginosa* to ciprofloxacin and norfloxacin, which is supported by a previous study demonstrating that chlorination can cause enrichment of antibiotic resistance bacteria and antibiotic resistance genes in the drinking water (Shi et al., 2013). However, after 64 h, all of the cells had already lost their cultivability and entered the “VBNC” state. Nevertheless, it was noted that long-term chlorination selected antibiotic-resistant *P. aeruginosa* strains. Hence, future research should concentrate on avoiding antibiotic-resistance microbes during chlorination, which is very important for a safe drinking water network.

Heterotrophic plate counts is considered to be a conventional parameter used for monitoring microbial inactivation during chlorination. However, the culture-dependent parameter is not suitable for microbial quality assessment in drinking water distribution network with residual chlorine (Nescerecka et al., 2014). Considerable underestimation of the bacterial population was detected when compared to the culture-independent methods (FCM and qPCR) (Chiao et al., 2014). In the current study, **Figure 3** shows a similar conclusion whereby the HPC counts were lower compared with the FCM counts, and the gap between the HPC and FCM counts became greater with increasing chlorine concentration. Similar patterns were reported for *E. coli* exposed to chlorine and UV-A light (Berney et al., 2006; Wang et al., 2010a). This may be due to the fact that some bacteria exposed to severe environmental stress (e.g., high chlorine concentration) lose the capacity for growth but retain their membrane integrity and enzymatic activity (Vital et al., 2012). When bacteria enter the VBNC state, remaining metabolically active and posing a potential threat to water quality (Bosshard et al., 2009; Wang et al., 2010a; Chiao et al., 2014), the traditional HPC method can no longer correctly enumerate the number of viable cells (Liu et al., 2015).

Among the different cell viability testing methods applied in this study, FCM and qPCR data showed clear correlations (**Supplementary Figure S3**). A significant linear correlation (*R*^2^ = 0.9) between qPCR (specific gene concentrations) and FCM (intact cell counts) methods was observed, demonstrating the two methods are complementary tools to characterize and monitor the efficiency of the chlorination processes. This result is consistent with a previous study that showed good correlation when comparing FCM with PMA-qPCR (Zacharias et al., 2015). Furthermore, FCM has been successfully applied to assess disinfection efficacy on bacteria (Ramseier et al., 2011; Nescerecka et al., 2014; Lee et al., 2016). Hence, it is evident that FCM is a proper tool in assessing accurate changes of bacterial cells during disinfection of drinking water.

## Conclusion

1. High chlorine exposure (0.47 mg/L, 1 h) could damage not only membrane integrity (ICC fingerprint) but also cellular DNA (TCC fingerprint), and DNA damage falls behind loss of membrane integrity. *P. aeruginosa* cells entered “VBNC” state under chlorine stress, which is not detectable using culture-dependent methods.
2. Free residual chlorine concentrations below 0.3 mg/L were found to reactivate *P. aeruginosa* in the low AOC medium system. Long time storage of water with insufficient chlorine levels may develop thriving bacteria that in turn influence water quality.
3. The long-term chlorine disinfection kinetics of *P. aeruginosa* shows three stages: an initial stage with relatively fast inactivation rate (*k* = 8.7 × 10^-3^), followed by a stationary stage (*k* = 3.0 × 10^-4^) and then a final stage with a slower inactivation rate than the initial stage (*k* = 2.7 × 10^-3^).
4. Long-term chlorination selected the antibiotic-resistant *P. aeruginosa*, although all antibiotic-resistant bacteria could be inactivated with subsequent continuous exposure to chlorine.

## Author Contributions

YW and MB designed and planned the experiments. GM and YS performed the experiments. GM, YS, YW, and MB analyzed the data. GM, YW, and MB wrote the manuscript.

## Conflict of Interest Statement

The authors declare that the research was conducted in the absence of any commercial or financial relationships that could be construed as a potential conflict of interest.

## Supplementary Material

FIGURE S1 Flow cytometric dot plots of *Pseudomonas aeruginosa* with SYBR Green I and stained with propidium iodide. **(A)** 0.38 mg/L chlorine without quenching solvent (1.27 ± 0.07 × 10^4^ cells/mL). **(B)** 0.38 mg/L chlorine with thiosulfate sodium quenching solvent (0.9 ± 0.03 × 10^4^ cells/mL).

FIGURE S2 Calibration curve of quantitative PCR with plasmid containing *gyrA* gene. The curve represents a range from 10^3^ to 10^9^ copies per reaction. Error bars show the standard deviation on triplicate measurements.

FIGURE S3 Comparison of the various culture-independent methods during chlorination. Clear correlations (*R*^2^ = 0.9) were observed between intact cells (FCM) and *gyrA* gene (qPCR).
