# Supplementary figures and images for: Long-Term Effects of Residual Chlorine on Pseudomonas aeruginosa in Simulated Drinking Water Fed With Low AOC Medium
Source: Front Microbiol. 2018 May 3;9:879. doi: 10.3389/fmicb.2018.00879 (PMC5943633; doi:10.3389/fmicb.2018.00879)

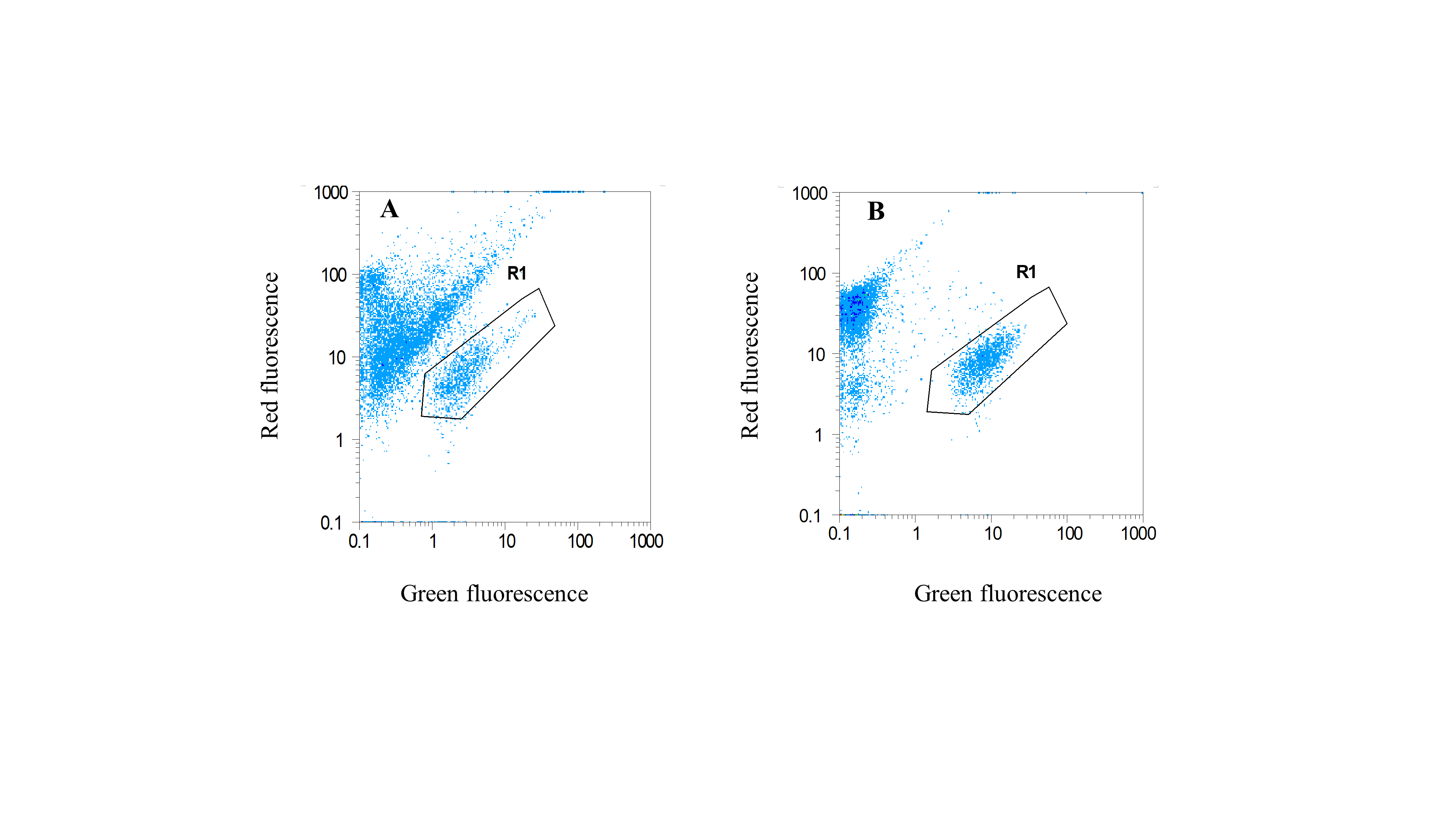

Supplement: FIGURE S1 — Flow cytometric dot plots of Pseudomonas aeruginosa with SYBR Green I and stained with propidium iodide. (A) 0.38 mg/L chlorine without quenching solvent (1.27 ± 0.07 × 10^4^ cells/mL). (B) 0.38 mg/L chlorine with thiosulfate sodium quenching solvent (0.9 ± 0.03 × 10^4^ cells/mL). [file Image_1.TIF]
